# Nobel Prize for physiology or medicine in 2024 for the discovery of microRNAs: small RNAs with fundamental roles in development and disease

**DOI:** 10.1007/s00424-024-03053-5

**Published:** 2024-12-11

**Authors:** Miguel Quévillon Huberdeau, Gunter Meister

**Affiliations:** 1 Regensburg Center for Biochemistry (RCB), Laboratory for RNA Biology, University of Regensburg, Regensburg, Germany; 2 Cluster for Nucleic Acid Therapeutics Munich (CNATM), Munich, Germany

MicroRNAs (miRNAs) are highly conserved small non-coding RNAs with fundamental roles in the regulation of gene expression. In humans, miRNAs have been intensively studied and links to developmental processes and almost all diseases have been found. Physiological processes such as the cardiovascular system, kidney function, or the immune system, for example, are orchestrated by miRNAs. The groundbreaking work in *Caenorhabditis elegans* already in 1993 by Gary Ruvkun and Victor Ambros [10] [6] opened the door for enlightening insights into this fundamental gene class and received the Nobel Prize in physiology or medicine in 2024 for these seminal discoveries. Their work also paved the way to target miRNAs for disease treatment [5].

miRNA genes are transcribed by RNA polymerase II as primary transcripts and go through several nuclear and cytoplasmic maturation steps. In the nucleus, the microprocessor complex with its RNase III enzyme Drosha generates a pre-miRNA hairpin that is further processed in the cytoplasm by Dicer to generate the mature, single-stranded miRNA strand [1, 9]. This strand is loaded onto an Argonaute protein and guides it to complementary sequences located on the 3ʹ untranslated region of target mRNAs, which are subsequently degraded. Ambros and Ruvkun realized that the *lin-14* gene contains multiple sequence elements that are complementary to the *lin-4* non-coding RNA, which are essential for controlling temporal expression of LIN-14 protein during *C. elegans* larval development [10]. The discovery of the second miRNA in *C. elegans*, encoded by *let-7* [8], and its conservation across a wide range of animals, including humans [7], set the stage for this rapidly expanding field of research. These findings are even more impressive, since miRNAs bind only partially complementary sequences on their target mRNAs. We now know that the functionally important miRNA “seed sequence” is only six to eight nucleotides long and most of such sites are still hardly predictable [1]. This powerful regulatory process shapes transcriptomes for cell identity and contributes to adaptation of gene expression profiles upon specific stimuli [3].

miRNA levels dynamically change when cells change their gene expression programs, for example when cells stop proliferating and start differentiating. miRNAs are tissue-specific and contribute to cell and organ identity by repressing genes with opposing functions. Consequently, each cell type, and thus tissue, is characterized by a very specific miRNA profile with only a handful of highly abundant “signature” miRNAs. Examples for such miRNAs are miR-122 in liver cells, miR-1 in heart cells, miR-155 in diverse cell types of the immune system, or miR-124 in neurons [3]. miRNAs important for cell proliferation during embryonic or tissue development are often reactivated in cancer. Prominent oncogenic miRNAs are members of the mR-17–92 cluster composed of miR-17, miR-18, miR-19, miR-20, and miR-92, which are frequently amplified in B cell lymphoma and many other cancer types [12]. A tumor suppressor miRNA is miR-34, which is activated by the transcription factor p53 and is thus less abundant in many cancers [4].

The groundbreaking work by Ambros and Ruvkun led to a back-then unseen explosion of research in RNA biology cumulating to more than 175,000 PubMed entries to date (search term “microRNA”). With all these results and given the importance of miRNAs for the development of diseases, strategies for therapeutic targeting of miRNAs are explored. Although the field is still in its infancy and miRNA-based drugs have not reached the market, many of such approaches are in clinical trials [5, 11]. Furthermore, circulating miRNAs that can be found in our blood systems seem to reflect specific disease states and are highly promising as biomarkers [2]. These exciting developments highlight the great potential of a research field that has been initiated by the two Nobel laureates more than 30 years ago.
